# Red blood cells in sports: effects of exercise and training on oxygen supply by red blood cells

**DOI:** 10.3389/fphys.2013.00332

**Published:** 2013-11-12

**Authors:** Heimo Mairbäurl

**Affiliations:** Medical Clinic VII, Sports Medicine, University of HeidelbergHeidelberg, Germany

**Keywords:** Hb-O_2_ affinity, blood gasses, 2,3-DPG, erythropoiesis, hypoxia inducible factor, ATP, NO, intravascular hemolysis

## Abstract

During exercise the cardiovascular system has to warrant substrate supply to working muscle. The main function of red blood cells in exercise is the transport of O_2_ from the lungs to the tissues and the delivery of metabolically produced CO_2_ to the lungs for expiration. Hemoglobin also contributes to the blood's buffering capacity, and ATP and NO release from red blood cells contributes to vasodilation and improved blood flow to working muscle. These functions require adequate amounts of red blood cells in circulation. Trained athletes, particularly in endurance sports, have a decreased hematocrit, which is sometimes called “sports anemia.” This is not anemia in a clinical sense, because athletes have in fact an increased total mass of red blood cells and hemoglobin in circulation relative to sedentary individuals. The slight decrease in hematocrit by training is brought about by an increased plasma volume (PV). The mechanisms that increase total red blood cell mass by training are not understood fully. Despite stimulated erythropoiesis, exercise can decrease the red blood cell mass by intravascular hemolysis mainly of senescent red blood cells, which is caused by mechanical rupture when red blood cells pass through capillaries in contracting muscles, and by compression of red cells e.g., in foot soles during running or in hand palms in weightlifters. Together, these adjustments cause a decrease in the average age of the population of circulating red blood cells in trained athletes. These younger red cells are characterized by improved oxygen release and deformability, both of which also improve tissue oxygen supply during exercise.

## Introduction

The primary role of red blood cells is the transport of respiratory gasses. In the lung, oxygen (O_2_) diffuses across the alveolar barrier from inspired air into blood, where the majority is bound by hemoglobin (Hb) to form oxy-Hb, a process called oxygenation. Hb is contained in the red blood cells, which, being circulated by the cardiovascular system, deliver O_2_ to the periphery where it is released from its Hb-bond (deoxygenation) and diffuses into the cells. While passing peripheral capillaries, carbon dioxide (CO_2_) produced by the cells reaches the red blood cells, where carbonic anhydrase (CA) in tissues and red blood cells converts a large portion of CO_2_ into bicarbonate (HCO^−^_3_). CO_2_ is also bound by Hb, preferentially by deoxygenated Hb forming carboxy-bonds. Both forms of CO_2_ are delivered to the lung, where CA converts HCO^−^_3_ back into CO_2_. CO_2_ is also released from its bond to Hb and diffuses across the alveolar wall to be expired.

**Figure 1:**
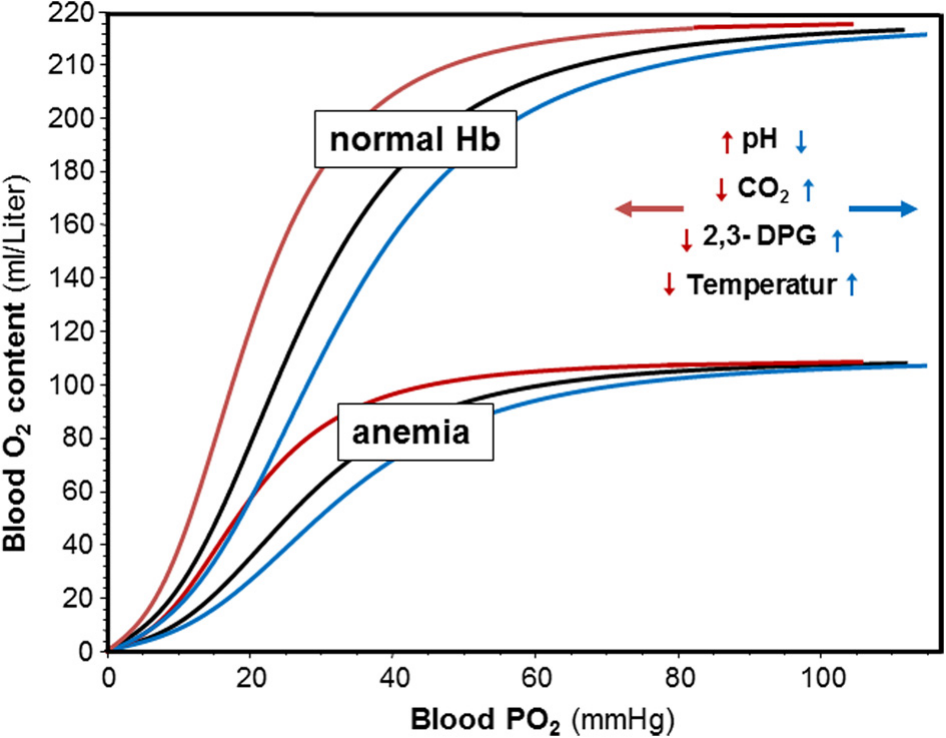
**Effects of hemoglobin concentration and pH, CO_2_, 2,3-DPG and temperature on blood oxygen content and on Hb-O_2_ affinity**. Oxygen dissociation curves (ODC) were calculated with the equation by Severinghaus (1979) using decreased, normal, and increased P_50_ values. Oxygen content was calculated from SO_2_ and normal and decreased hemoglobin concentrations assuming that 1 g H binds 1.34 ml of O_2_. The insert indicates that an increase in pH, and a decrease in CO_2_, 2,3-DPG and temperature shifts the ODC to the left (red arrows and curves), whereas acidosis and increased CO_2_, 2,3-DPG and temperature shift the ODCs to the right.

The biological significance of O_2_ transport by Hb is well-illustrated by anemia where decreased Hb also decreases exercise performance despite a compensatory increase in cardiac output (Ledingham, 1977; Carroll, 2007), and by improved aerobic performance upon increasing total Hb (Berglund and Hemmingson, 1987). The O_2_ dissociation curves in Figure 1 indicate the advantage of normal vs. anemic Hb showing that the O_2_ content in blood varies with the Hb concentration in blood at any given O_2_ partial pressure (PO_2_). Not only its amount but also the functional properties of Hb affect performance. This is illustrated by the observation that an increased Hb-O_2_ affinity favors O_2_ loading in the lung and survival in an hypoxic environment (Eaton et al., 1974; Hebbel et al., 1978), whereas a decreased Hb-O_2_ affinity favors the release of O_2_ from the Hb molecule in support of oxidative phosphorylation when the ATP demand is high, such as in exercising skeletal muscle (for a recent review see Mairbäurl and Weber, 2012).

Despite O_2_ transport, red blood cells fulfill a variety of other functions, all of which also may improve exercise performance. Likely the most important one is the contribution of red blood cells in buffering changes in blood pH by transport of CO_2_ and by binding of H^+^ to hemoglobin. Red blood cells also take up metabolites such as lactate that is released from skeletal muscle cells during high intensity exercise. Uptake into red blood cells decreases the plasma concentration of metabolites. Finally, red blood cells seem to be able to decrease peripheral vascular resistance by releasing the vasodilator NO (Stamler et al., 1997) and by releasing ATP which stimulates endothelial NO formation causing arteriolar vasodilation and augments local blood flow (Gonzalez-Alonso et al., 2002).

This review summarizes the mechanisms by which red blood cells warrant O_2_ supply to the tissues with special emphasis on O_2_ transport to exercising muscle.

## Oxygen affinity of hemoglobin

A major mechanism optimizing O_2_ transport by hemoglobin is the change in Hb-O_2_ affinity. Changes are very fast and actually occur while red blood cells pass through blood capillaries. Effects of altered Hb-O_2_ affinity on O_2_ transport are independent of Hb concentration and total Hb mass in circulation and thus add to the adjustment by changes in erythropoiesis.

The intrinsic O_2_-affinity of hemoglobin is very high (Weber and Fago, 2004). Therefore, allosteric effectors are required that decrease Hb-O_2_ affinity allowing unloading of O_2_ from the Hb molecule. The major allosteric effectors modulating Hb-O_2_ affinity *in vivo* in human red blood cells are organic phosphates such as 2,3-diphosphoglycerate (2,3-DPG) and adenosine triphosphate (ATP), H^+^ and CO_2_, and Cl^−^. A direct role of lactate, which accumulates during exercise, on Hb-O_2_-affinity is less clear and may be due to a small effect on the Cl^−^ binding by Hb and on carbamate formation (reviewed in (Mairbäurl and Weber, 2012)). Indirect effects of lactate may be caused by affecting the Cl^−^ concentration and by the uptake of H^+^ together with lactate mediated by MCT-1 (Deuticke, 1982). Another modulator of Hb-O_2_ affinity relevant to exercise is a change in body temperature (Dill and Forbes, 1941; Mairbäurl and Humpeler, 1980). Figure 1 shows that at any Hb concentration, acidosis, and an increase in CO_2_ and 2,3-DPG decrease Hb-O_2_ affinity. Changes in Cl^−^ are small *in vivo* and are therefore not shown on the graph. Also an increase in temperature decreases Hb-O_2_ affinity. These changes shift the ODC to the right showing graphically that the O_2_ saturation of Hb (SO_2_) is decreased at any given PO_2_. In contrast, alkalosis, a decrease in CO_2_, 2,3-DPG, and temperature increase Hb-O_2_ affinity to increase SO_2_ at a given PO_2_.

The physiological significance of an increased Hb-O_2_ affinity is an improved O_2_ binding by Hb when the PO_2_ is low. It is therefore of significance for people exposed to hypoxic environments, where it prevents exaggerated arterial desaturation. A decrease in Hb-O_2_ affinity improves O_2_ delivery to cells with a high O_2_ demand such as in exercising muscle (see below).

A simple approach to estimate the SO_2_ from PO_2_ and vice versa has been published by Severinghaus (1979). The formula was derived from a best fit model of the standard oxygen dissociation curve with an error of SO_2_ of 0.26% within the physiological range of PO_2_. The standard half saturation pressure of O_2_ (P_50_value) was given as 26.86 mmHg at a plasma pH = 7.4 and 37°C; S is fractional saturation.

$$\begin{array}{l} \text{S}=100\times {\left(\left({\left(\text{P}{\text{O}}_{2}^{3}+150\times \text{P}{\text{O}}_{2}\right)}^{-1}\times 23400\right)+1\right)}^{-1}\text{or}\\ \text{ln}\;\text{P}{\text{O}}_{2,\text{st}}=0.385\times \text{ln}\;({\text{S}}^{-1}-1)+3.32-{\left(72\times \text{S}\right)}^{-1}-0.17\times {\text{S}}^{6} \end{array}$$

Based on a model proposed by Roughton and Severinghaus (1973), Okada et al. (1977) published a modification of this formula that allows estimating changes in P_50_ by altered pH, temperature (T; °C), base excess (BE; mEq/Liter), and 2,3-DPG (DPG; molar ration of 2,3-DPG to Hb) with accuracies of P_50_ values and SO_2_ of ± 2.5 and ± 5%, respectively.

$$\begin{array}{l} \Delta \log_{50}=0.48\times (7.4-{\text{pH}}_{\text{plasma}})+0.024\times (\text{T}-37)\\ \;+0.0013\times \text{BE}+0.135\times \text{DPG}-0.116, \end{array}$$

After correction of P_50_ using this equation to obtain P_50,actual_, adjusted PO_2_ (PO_2,actual_) values can be calculated (Severinghaus, 1979) as

$${\text{PO}}_{2,\text{actual}}={\text{PO}}_{2,\text{std}}\times \frac{{\text{P}}_{50,\text{actual}}}{26.86}$$

Then the “Severinhaus-equation” can be used to calculate S from the new PO_2_ to obtain complete ODCs. A more detailed description of the magnitude of changes in Hb-O_2_ affinity by allosteric effectors, temperature, and other molecules alone as well as their interactions is reviewed in (Mairbäurl and Weber, 2012).

### Hb-O_2_ affinity during exercise

During exercise the increased demand for oxygen is met by increasing muscle blood flow (Laughlin et al., 2012) and by improved O_2_ unloading from Hb achieved by decreasing Hb-O_2_ affinity (Mairbäurl, 1994). It is obvious that a decreased Hb-O_2_ affinity, if occurring systemically—i.e., in all red blood cells in circulation—will compromise arterial O_2_ loading of Hb in the lung. It would thus be advantageous if adjustments in Hb-O_2_ affinity occurred locally to serve both functions, oxygenation in the lung and deoxygenation in peripheral blood capillaries. Thus, Hb-O_2_ affinity should be low while red blood cells pass through tissues with a high O_2_ demand, and should be increased when red blood cells return to the lung. This is in fact what happens because of distinct differences in pH, CO_2_ and temperature between the lung and capillaries in working muscles. No changes in 2,3-DPG, one of the major allosteric effectors of Hb-O_2_ affinity (Benesch and Benesch, 1967), during exercise tests have been observed (Mairbäurl et al., 1986), because 2,3-DPG changes are slow and require adjustments of the glycolytic rate in red blood cells. However, elevated 2,3-DPG has been found after training (Böning et al., 1975; Braumann et al., 1982; Mairbäurl et al., 1983; Schmidt et al., 1988). It might be considered beneficial for O_2_ unloading during exercise because it increases the effect of acidosis (Bohr effect) on Hb-O_2_ affinity (Bauer, 1969). The elevated 2,3-DPG in trained individuals might be a consequence of the stimulated erythropoiesis, which decreases red blood cell age (Mairbäurl et al., 1983). Young red blood cells have an increased metabolic activity (Seamen et al., 1980; Rapoport, 1986), higher 2,3-DPG, and a lower Hb-O2-affibity than senescent red blood cells (Haidas et al., 1971; Mairbäurl et al., 1990).

#### O_2_unloading to exercising muscle

**Figure 2:**
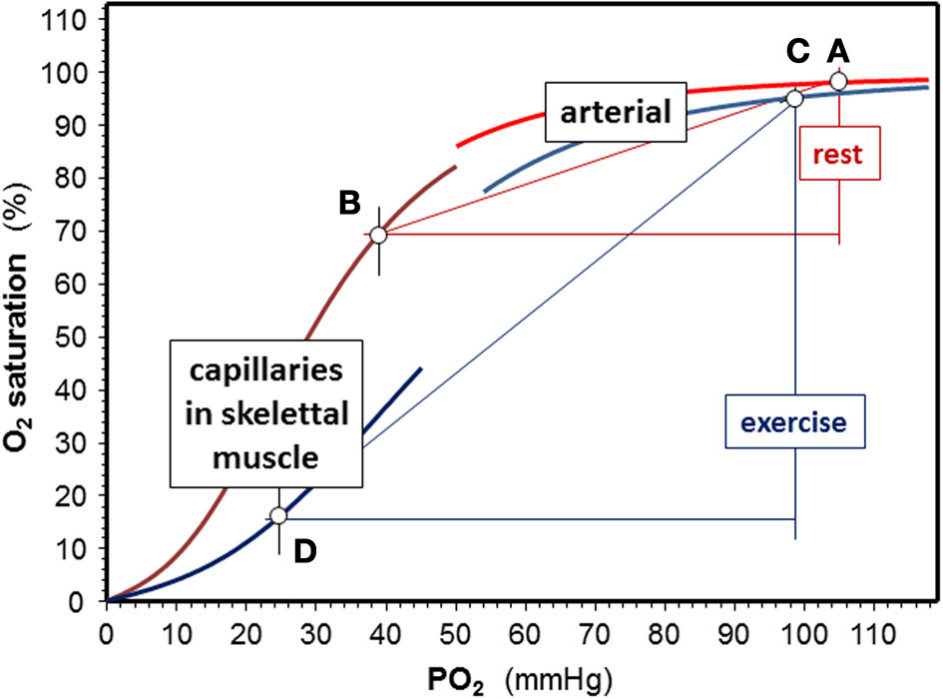
**Effects of exercise on Hb-O_2_ affinity**. Shifts of the O_2_-dissociation curves are calculated for an arterial pH = 7.4, a capillary pH = 7.3, and temperature is 37°C rest. Values used to calculate oxygen dissociation curves (ODC) for exercise were an arterial pH = 7.15 at 38.5°C, a capillary pH = 7.0 and temperature = 41°C in working muscle using the equation giving in the text using data from exercise tests (Sun et al., 2000). At rest, assuming a venous PO_2_ = 40 mmHg, SO_2_ decreases by 28% (points **A** and **B**), while extraction nearly triples in exercise conditions (delta SO_2_ = 79%) assuming a venous PO_2_ = 25 mmHg during exercise (points **C** and **D**).

Exercising muscle cells release H^+^, CO_2_, and lactate into blood capillaries, and there is also a higher temperature in working muscle than in inactive tissues. Blood entering capillaries of exercising muscles is acutely exposed to these changes, which causes a rapid decrease in Hb-O_2_ affinity. P_50_ values of ~34–48 mmHg can be estimated from changes in blood gasses (provided e.g., in Sun et al., 2000). Temperature increases from 37°C at rest to 41°C during exercise. Because there is a continuous change in blood composition by admixture of metabolites as new blood enters a capillary, P_50_ values are lower at the arterial side of the capillaries than at their venous end (Mairbäurl and Weber, 2012) causing an enormous rightward shift of the ODC within the capillaries that increases unloading of O_2_ from Hb considerably (Berlin et al., 2002). This is also demonstrated by the extensive shift to the right of the ODC in capillary blood in exercise conditions relative to rest (Figure 2; points D and B, respectively). Trained individuals have a higher Bohr effect at low SO_2_ probably due to elevated 2,3-DPG (Böning et al., 1975; Braumann et al., 1982; Mairbäurl et al., 1983), which might cause an even greater increase in the arterio-to-venous O_2_ difference.

#### Arterial O_2_ loading

On its way from working muscle to the lung the concentrations of H^+^ and CO_2_ in blood are decreased by admixture of blood coming from inactive muscle and other organs. CO_2_ decreases in alveolar capillaries due to alveolar gas exchange, which further alkalinizes the blood. Thus, the effects of these metabolites on Hb-O_2_ affinity are attenuated in the lung relative to working muscle. Also the temperature is lower in the lung than in working muscle. Nevertheless, normal values of Hb-O_2_ affinity are not restored completely during intensive exercise, which is indicated by the slight shift to the right of the ODC in the exercise conditions relative to the resting situation (Figure 2; points A and C). The magnitude of the deviation depends on the active muscle mass and exercise intensity. From blood gas data during exercise reported by Wasserman and colleagues (Sun et al., 2000) it can be estimated that the half saturation tension of O_2_ (P_50_ value) might increase from about ~27 mmHg at rest to 34 mmHg in arterial blood during heavy exercise. This decrease in Hb-O_2_ affinity impairs arterial O_2_ loading and decreases arterial SO_2_ from ~97.5% at rest to ~95% during high intensity exercise. An increased 2,3-DPG in trained individuals might further decrease arterial SO_2_ (Mairbäurl et al., 1983). Adding to the effect of decreased Hb-O_2_ affinity, SO_2_ decreases further because of diffusion limitation by the shortened contact time when cardiac output is high (Dempsey and Wagner, 1999; Hopkins, 2006), which might even be enhanced when exercise is performed in hypoxia (Calbet et al., 2008).

When comparing the effects of acid metabolites and the increased body temperature during exercise on Hb-O_2_ affinity in arterial and muscle capillary blood it is evident that the changes are much greater in working muscle than in the lung. Thus, the greatly increased amount of O_2_ unloaded from Hb relative to rest easily compensates for arterial desaturation during exercise.

## Oxygen transport capacity

Whereas only 0.03 ml O_2_^*^ L^−1^^*^ mmHg^−1^ PO_2_ at 37°C can be transported in blood in physical solution, one gram of Hb can bind ~1.34 ml of O_2_. Thus, the presence of a normal amount of Hb per volume of blood increases the amount of O_2_ that can be transported about 70-fold, which is absolutely essential to meet the normal tissue O_2_ demand. It is therefore apparent that an increased amount of Hb also increases the amount of O_2_ that can be delivered to the tissues (Figure 1). In fact, the O_2_ transport capacity was found to correlate directly with aerobic performance as can be seen from an increase in performance after infusion of red blood cells (Berglund and Hemmingson, 1987) and by the strong correlation between total Hb and maximal O_2_ uptake (VO_2,max_) in athletes (for review see Sawka et al., 2000; Schmidt and Prommer, 2010). Calbet et al found that acute manipulations of the O_2_ carrying capacity also vary performance (Calbet et al., 2006). Thus, it is a clear advantage for aerobic athletic performance to have a high O_2_ transport capacity.

Parameters required to evaluate O_2_ transport capacity are the Hb concentration in blood (cHb) and hematocrit (Hct), as well as total Hb mass (tHb) and total red blood cell volume (tEV) in circulation. cHb and Hct are easy to measure with standard hematological laboratory equipment. Together with SO_2_ they indicate the amount of O_2_ that can be delivered to the periphery per unit volume of cardiac output. tHb and tEV indicate the total amount of O_2_ that can be transported by blood. A large tHb and tEV allows redirecting O_2_ to organs with a high O_2_ demand while maintaining basal O_2_ supply in less active tissues. Because they are affected by changes in plasma volume (PV) cHb and Hct allow no conclusion on tHb and tEV, respectively.

Results on cHb, Hct and red blood cell count in athletes and their comparison with values obtained in healthy, sedentary individuals are conflicting due to the fact that red blood cell volume and PV change independently and due to the many factors affecting each of these parameters (see below). Establishing normal values for tHb and tEV for athletes is hampered further by the possibility of use of means to increase aerobic capacity such as blood and erythropoietin (EPO) doping.

### Hematocrit in athletes

Many but not all studies show lower Hct in athletes than in sedentary controls (Broun, 1922; Davies and Brewer, 1935; Ernst, 1987; Sawka et al., 2000). However, several studies also report higher than normal Hct. A highly increased Hct increases blood viscosity and increases the workload of the heart (El-Sayed et al., 2005; Böning et al., 2011). It therefore bears the risk of cardiac overload.

Many studies showed that Hct tended to be lower in athletes than in sedentary individuals (Broun, 1922; Davies and Brewer, 1935; Remes, 1979; Magnusson et al., 1984; Selby and Eichner, 1986; Ernst, 1987; Weight et al., 1992). This was verified by Sharpe et al. (2002) in the course of establishing reference Hct and Hb values for athletes. The found that out of ~1100 athletes from different countries 85% of the female and 22% of the male athletes had Hct values below 44%. A tendency for an inverse correlation of Hct with training status, indicated by VO_2,max_, was also shown (Heinicke et al., 2001). However, a small proportion of sedentary controls and athletes has higher than normal Hct. Sharpe et al. (2002) found that 1.2% of all females and 32% of all males in their study had an Hct > 47%. When following female and male elite athletes and controls over a study period of 43 months Vergouwen (Vergouwen et al., 1999) found 6 males controls and 5 males athletes with a Hct > 50% and 5 females controls but no female athletes with a Hct > 47%.

#### Hematocrit during exercise

Changes in Hct occur rapidly. Hct increases during exercise due to a decrease in PV when fluid replacement during exercise is insufficient (Costill et al., 1974). There is fluid loss due to sweating, a shift of plasma water into the extracellular space due to the accumulation of osmotically active metabolites, and filtration as a consequence of an increased capillary hydrostatic pressure (Convertino, 1987). The resultant increase in plasma protein increases oncotic pressure and thus moderates fluid escape (Harrison, 1985). Changes appear less pronounced during swimming than running exercise, where immersion and the re-distribution of blood volume seem to cause shifts in PV independent of volume regulatory hormones (Böning et al., 1988). An increase in hematocrit due to catecholamine-induced sequestration of red blood cells from spleen is unlikely in humans but has been found in other species (Stewart and McKenzie, 2002).

#### Long-term changes of hematocrit

In a recent review, Thirup (2003) reports a within-subject variability of ~3% when reviewing 12 studies on more than 600 healthy, non-smoking, mostly sedentary individuals, and when measurements were repeated in sampling intervals ranging from days to ~2 months. Sawka et al. summarized data from 18 investigations and found that PV and blood volume increased rapidly after training sessions, whereas red cell volume remained unchanged for several days before it began to increase indicating that Hct values were decreased for several days (Sawka et al., 2000). The magnitude of Hct change seems to depend on exercise intensity during training sessions and the type of exercise (strength vs. endurance; for review see Hu and Lin, 2012). A few weeks after the training intervention a new steady state had established, and Hct had returned to pre-training values (Sawka et al., 2000). The post-training increase in PV and the increased PV in highly trained athletes (e.g., Hagberg et al., 1998; Sawka et al., 2000; Heinicke et al., 2001; Schumacher et al., 2002) is likely caused by aldosterone dependent renal Na^+^ reabsorption, and by water retention stimulated by elevated antidiuretic hormone in compensation for the water loss during individual training sessions (Costill et al., 1976; Milledge et al., 1982).

There appear to be quite large seasonal variations in Hct (relative change up to 15%) with lower values in summer than in winter that might result in season-to-season changes from ~42% in summer and 48% in winter as found among several thousand study participants. Seasonal changes depend on climatic effects with larger differences in countries closer to the equator (Thirup, 2003). Studies of seasonal changes in Hct of athletes are sparse but indicate that Hct might be decreased by another 1–2% in summer by addition of a training effect.

The decreased Hct in athletes has been termed “sports anemia.” For a long time it had been explained with increased red blood cell destruction during exercise and thus appeared to be the same phenomenon as the well-known march hemoglobinuria (Broun, 1922; Kurz, 1948; Martin and Kilian, 1959). Intravascular destruction of red blood cells occurs at shear stresses between 1000 and 4000 dyn/cm^2^ (Sutera, 1977; Sallam and Hwang, 1984), values well above physiological values at rest (Mairbäurl et al., 2013). It is related to the intensity and the kind of exercise (Yoshimura et al., 1980; Miller et al., 1988). Foot strike in runners has been the most often reported reason for intravascular hemolysis (Telford et al., 2003), which can be prevented by good shoe cushioning (Yoshimura et al., 1980; Dressendorfer et al., 1992). It also occurred during mountain hiking (Martin et al., 1992), in strength training (Schobersberger et al., 1990), karate (Streeton, 1967), in swimmers (Selby and Eichner, 1986; Robinson et al., 2006), basketball, Kendo-fencing, and in drummers (Schwartz and Flessa, 1973; Nakatsuji et al., 1978). Running exercise has been found to increase plasma hemoglobin from ~30 mg/liter at rest to ~120 mg/liter indicating that about 0.04% of all circulating red blood cells were lyzed (Telford et al., 2003). Exercise had been shown to alter red blood cell membrane appearance in correlation with elevated haptoglobin (Jordan et al., 1998). Senescent red blood cells may be particularly prone to exercise induced intravascular hemolysis as indicated by a decreased mean red blood cell buoyant density and a density distribution curve that was skewed toward younger, less dense cells in trained individuals indicated by increased levels of pyruvate kinase activity, 2,3-DPG and P_50_, higher reticulocyte counts (Mairbäurl et al., 1983). Other possible reasons for “sports anemia” under discussion are nutritional aspects such as insufficient protein intake and altered profile of blood lipids (for review see Yoshimura et al., 1980), and iron deficiency (Hunding et al., 1981).

### Total hemoglobin mass (tHb) and total red blood cell volume (tEV)

As indicated above, PV is prone to acute changes, whereas changes in total red blood cell mass (or volume) are slow due to slow rates of erythropoiesis (Sawka et al., 2000). Therefore, total hemoglobin and/or red blood cell volume has to be measured in addition to cHb and Hct to obtain a reliable measure of the oxygen transport capacity. Several methods have been applied to determine these parameters.

Grehant and Quinquard (1882) were the first to describe blood volume measurements by use of carbon monoxide (CO)-rebreathing. This method is based on the much higher affinity of Hb to CO than to O_2_ (for review see Mairbäurl and Weber, 2012), which allows using CO in an indicator dilution method. It has been used to measure the fraction of blood mass relative to body mass by Arnold et al. (1921). The technique has been improved considerably by Sjostrand by advancing the method to estimate carboxy-hemoglobin (Sjostrand, 1948). To date CO rebreathing or inhalation has been further improved (Godin and Shephard, 1972; Schmidt and Prommer, 2005). MCHC is then used to calculate tEV, and Hct to estimate total blood volume. Total red blood cell volume can be determined directly after injection of ^99m^Tc-labeled red blood cells (Thomsen et al., 1991). By indirect means, total red blood cell volume can also be calculated from Hct after measuring the PV using Evans blue (T-1824), which binds to albumin, and by injection of ^125^iodine-labeled albumin. Several of these methods have been compared by Thomsen et al. (1991) who reported a correlation of *r* = 0.99 between PV measured by ^125^I-albumin and Evans blue, and showed that PV calculated from measuring tEV with labeled red blood cells was about 5–10% lower than that from labeling albumin.

Applying these techniques Kjellberg et al. found that trained individuals had increased tHb (Kjellberg et al., 1949), a result that has been confirmed many times thereafter both by comparing groups of individuals with different training status and by measuring tEV before and after prolonged training periods (for a recent review see Sawka et al., 2000). Schmidt and Prommer summarized recently that different training modalities vary in their effects on tHb, where they put the main emphasis on training in hypoxia (Schmidt and Prommer, 2008). In summary, these studies show that an increase in tHb by 1 g achieved e.g., by administration of erythropoietin, increased VO_2,max_ by ~3 ml/min (Parisotto et al., 2000; Schmidt and Prommer, 2008). From the correlation shown by Heinicke et al. (2001) it can be derived that an increase in 1 g of tHb per kg body weight (g/kg) increased VO_2,max_ by ~5.8 ml/min/kg, where non-athletes (though with a rather high VO_2,max_ of 45 ml/min/kg) had a tHb of 11 g/kg and their best athletes (average VO_2,max_ = 71.9 ml/kg) had a tHb of 14.8 g/kg (Heinicke et al., 2001). Their findings fit well to the results reported by Kjellberg, who found a 37% higher tHb in elite athletes than in untrained individuals (Kjellberg et al., 1949). Schmidt and Prommer (2008) combined results from several of their studies and found a change in VO_2,max_ of 4.2 ml/min/kg in males and of 4.4 ml/min/kg in females per change in tHb of 1 g/kg with very high correlation coefficients (*r*~ 0.79), whereas there was no correlation between VO_2,max_ and Hb or Hct. However, there are also reports on a lack of difference in tHb between sedentary and trained individuals (Green et al., 1991). As mentioned above all these studies bear the burden of uncertainty that athletes may have taken measures to increase performance, which makes it difficult to establish “normal values” of tHb and tEV for athletes.

Different duration of exercise training (weeks vs. months) appear to explain the diverging results in the studies on tHb and training. Sawka et al. (2000) found no increase when training lasted less than 11 days. Also most studies on 4–12 months of training showed no or only small effects; their own longitudinal study on “leisure sportsmen” resulted in an increase in tHb by ~6% in the course of a 9-month endurance training (summarized in Schmidt and Prommer, 2008) indicating that adjustments of tHb and tEV by training are slow, and that a pronounced increase may require several years of training.

Sedentary high altitude residents have an increased tHb in comparison to their low altitude counterparts, where blood volume has been found to be increased from ~80 to ~100 ml/kg (Hurtado, 1964; Sanchez et al., 1970). Results on sojourners to high altitude indicate that, similar to training, the increase in tHb and blood volume is also slow requiring weeks to months of high altitude exposure. At high altitude, the increase may be masked by a decrease in PV (Reynafarje et al., 1959). Therefore, a short-term stay at moderate and high altitude will not increase tHb and tEV (Myhre et al., 1970). A summary of 14 different studies Sawka et al. (2000) shows that several studies found no change in tEV upon ascent whereas some did, and explained discrepancies with the difference in the duration of exposure to high altitude. A gain in tEV between 62 and 250 ml/week was found when the sojourn lasted about 3 weeks.

Based on the raise in tEV upon ascent to high altitude and by training in normoxia it was concluded that effects of training and high altitude exposure on tHb might be additive, and that training at simulated altitude or by ascent to moderate or high altitude should cause an even further increase than training in normoxia. However, results are inconsistent ranging from no effect (Svedenhag et al., 1997; Friedmann et al., 1999) to a pronounced increase after 3–4 weeks of training at altitudes between 2100 and 2400 m (Levine and Stray-Gundersen, 1997; Friedmann et al., 2005; Heinicke et al., 2005). Lack of effects has in part been explained with lower training intensities at high than at low altitude, which is due to the decrease in performance with increasing altitude (Cerretelli and DiPrampero, 1985). Several strategies have been developed aimed at improving the training efficiency while still “consuming” adjustments to hypoxia, one being the “sleep-high-train-low” protocol. Current concepts and concerns are reviewed in (Richalet and Gore, 2008; Stray-Gundersen and Levine, 2008; Robach and Lundby, 2012). Results are unclear, and often show no effect on tHb [e.g., in a well-designed, Placebo-controlled study by Siebenmann et al. (2012)]. A thorough analysis reveals that more than 14h per day of exposure to hypoxia seem to be required to attain a detectible increase in tHb and tEV (analysis in Schmidt and Prommer, 2008).

#### Control of erythropoiesis

It has been recognized by Bert (1882) that live at high altitude corresponds with increased hemoglobin, and later that Hct, Hb, and tHb are increased (Reynafarje et al., 1959; Hurtado, 1964; Sanchez et al., 1970), which was later recognized to be associated with elevated levels of erythropoietin (Mirand and Prentice, 1957; Scaro, 1960; Siri et al., 1966). The elevated tEV is thought to compensate for the decreased arterial O_2_-content when the inspired PO_2_ is low. Stimulation of vascularization by the vascular endothelial growth factor, VEGF, is another means warranting tissue O_2_ supply in chronic hypoxia (for review see e.g., Marti, 2005). Both processes depend on sensing hypoxia within typical target cells and specific signaling pathways that adjust the expression of specific genes.

One such oxygen dependent mechanism is the control of expression by hypoxia inducible factors, HIF (Semenza, 2009). Active HIF consists of alpha and beta subunits. The beta subunit (HIF-β, also called ARNT) is expressed constitutively and is not directly affected by oxygen levels (Semenza, 1999). There are several isoforms of the alpha subunit, where HIF-1α seems to mainly control metabolic adjustments such as glycolysis (Hu et al., 2003), and HIF-2α has been identified as the major regulator of erythropoiesis (Scortegagna et al., 2005; Gruber et al., 2007). In hypoxia, the hydroxylation of HIF-alpha subunits by prolyl-hydroxylases (PDH) is prevented due to the lack of O_2_ required as a direct substrate, which then prevents the hydroxylation-dependent poly-ubiquitinylation by the Van Hippel-Lindau tumor suppressor pVHL-E3 ligase and subsequent proteasomal degradation (Schofield and Ratcliffe, 2004) resulting in increased protein levels of HIF alpha subunits. Upon stabilization, alpha subunits enter the nucleus, where they dimerize with HIF-β. The dimer binds to a specific base sequence in the promoter region of genes called hypoxia response element, HRE, to induce the expression of genes (for recent reviews see (Semenza, 2009; Haase, 2010)). Besides stabilization, HIF-alpha subunits are also controlled at the transcriptional level (Görlach, 2009; Semenza, 2009).

In his review Haase (2010) nicely summarizes the experiments that led to the conclusion that HIF-2α is the major regulator of EPO production in liver (fetal) and kidney (adults), but that there are also a variety of different direct and indirect mechanisms. As shown in the scheme provided by Semenza (2009), although at that time related to actions of HIF-1α rather than HIF-2α, it can be seen that hypoxia controlled gene expression regulates not only the expression of EPO but also the expression of proteins whose action is a prerequisite for erythropoiesis such as EPO-receptors, iron transporters mediating intestinal iron reabsorption, and transferrin and transferrin receptors required for iron delivery to peripheral cells.

In the adult, the oxygen sensor controlling EPO production is in the kidney, where the cells producing EPO have been shown to be peritubular fibroblasts in the renal cortex (Maxwell et al., 1993; Eckardt and Kurtz, 2005). EPO production can be induced by two kinds of hypoxia: one is a decreased PO_2_ in the kidney and in other tissues while the hemoglobin concentration is normal such as in hypoxic hypoxia. The other is called anemic hypoxia, where the hemoglobin concentration is decreased and but arterial PO_2_ is normal resulting in a decreased venous PO_2_ (Eckardt and Kurtz, 2005). There appears no difference in the effectiveness to produce EPO between these two situations. A mixture of these conditions might be a situation causing a decreased blood flow to the kidney at normal PO_2_ and hemoglobin concentration, which should also result in decreased capillary and venous PO_2_. The exact mechanisms controlling EPO production by the fibroblasts is not fully understood but appears to involve hypoxia-dependent recruitment of fibroblasts located in juxta-medullary and cortical regions (Eckardt et al., 1993).

EPO released into blood has many functions other than stimulating erythropoiesis (for review see Sasaki, 2003). In the bone marrow EPO binds to EPO receptors on progenitor cells in erythroblastic islands (Chasis and Mohandas, 2008), where it stimulates proliferation and prevents apoptotic destruction of newly formed cells (Lee and Percy, 2010). This increases the amount of red blood cells released from the bone marrow per time resulting in increased tEV when the rate of release exceeds red blood cell destruction (see above, sports anemia).

#### Effects of exercise and training on erythropoiesis

The increased tHb and tEV in trained athletes indicates that exercise stimulates erythropoiesis. An additional marker is the elevation of reticulocytes counts which can be observed within 1–2 days (Schmidt et al., 1988) after endurance (Convertino, 1991) and strength training units (Schobersberger et al., 1990). Despite apparent effects of single training units on red blood cell production several studies show that reticulocyte counts in athletes are not much different from sedentary controls (Lombardi et al., 2013) and values appear quite stable over years (Banfi et al., 2011; Diaz et al., 2011). There is, however, significant variation of reticulocyte counts in athletes during the year showing in general higher reticulocyte counts at the beginning of a season but lower values after intensive training sessions, competitions, and at the end of a season (Banfi et al., 2011). Nevertheless, markers of pre-mature forms of reticulocytes are increased in athletes, which is indicative of stimulated bone marrow (Diaz et al., 2011; Jelkmann and Lundby, 2011).

Whereas the control of erythropoiesis in hypoxic and anemic hypoxia is well-understood, the signals stimulating erythropoiesis upon training in normoxia are unclear. Exposure to hypoxia causes a fast increase in EPO (Eckardt et al., 1989), but no or only minor changes in EPO have been observed after exercise of different modalities in untrained and trained individuals (Schmidt et al., 1991; Bodary et al., 1999), whereas the time course of change in reticulocyte count is similar to effects of high altitude (Schmidt et al., 1988; Mairbäurl et al., 1990). The higher reticulocyte counts, a decreased mean red blood cell buoyant density and mean corpuscular hemoglobin concentration, and increased levels of other markers of a decreased mean red blood cell age (higher 2,3-DPG and P_50_, higher red blood cell enzyme activities and creatine) have been found in peripheral blood from trained individuals (Mairbäurl et al., 1983; Schmidt et al., 1988), which are all indicators of an increased red blood cell turnover (Schmidt et al., 1988; Smith, 1995) and thus stimulated erythropoiesis. These newly formed red blood cells ease the passage of blood through capillaries because of a higher membrane fluidity and deformability of (Kamada et al., 1993).

Arguments for hypoxia as the relevant trigger for exercise induced erythropoiesis are sparse, and are at best indirect. Even during heavy exercise there is only a small decrease in arterial PO_2_ (see chapter 2, arterial O_2_ loading) that by itself will barely be sufficient to cause relevant renal EPO production. There is, however, a considerable decrease in renal blood flow with increasing exercise intensity that decreases renal O_2_ supply (for an excellent review on splanchnic blood flow regulation in exercise see Laughlin et al., 2012). The O_2_ supply to renal tubules might be further decreased, because renal cortical arteries and veins run parallel allowing exchange diffusion of O_2_ that may cause arterial deoxygenation. PO_2_ in cortical veins is low because of the high oxygen consumption required for Na^+^ and water reabsorption of renal cortical epithelial cells (Eckardt and Kurtz, 2005). It can therefore be speculated that the decreased flow during exercise further decreases renal cortical PO_2_ to a level causing significant hypoxia of the peritubular, EPO producing fibroblasts during exercise, and that this effect is aggravated as exercise intensity increases. Interestingly, training attenuates the decrease in renal blood flow, which seems more pronounced following endurance than high-intensity interval sprint training in rats (Musch et al., 1991; Padilla et al., 2011), which might explain the weak erythropoietic response in highly trained athletes.

A variety of humoral factors known to affect erythropoiesis also change during exercise. Androgens are long known for their stimulatory effect on erythropoiesis by stimulation of EPO release, increasing bone marrow activity, and iron incorporation into the red cells, which is best indicated by polycythemia as a consequence of androgen therapy (Shahidi, 1973; Shahani et al., 2009). Endurance exercise and resistance training cause a transient increase in testosterone levels in men and women (Hackney, 2001; Enea et al., 2009). Post-exercise values vary with exercise intensity in both genders. Interestingly, post-exercise testosterone levels also directly change with mood (win vs. loss), which seems more pronounced in men than women (for review see Shahani et al., 2009).

Stress hormones such as catecholamines and cortisol stimulate the release of reticulocytes from the bone marrow and possibly also enhance erythropoiesis (Dar et al., 2011; Hu and Lin, 2012). Erythropoiesis is also stimulated by growth hormone and insulin-like growth factors (Kurtz et al., 1988; Christ et al., 1997) which also increase during exercise (Hakkinen and Pakarinen, 1995; Schwarz et al., 1996).

## Hemorheology

Hematocrit not only affects the amount of O_2_ that can be carried per volume of blood but also affects the rheological properties of blood. Due to its composition of plasma and blood cells it behaves as a non-Newtonian fluid, whose inner viscosity is affected by the shear forces and is determined by the concentration of plasma proteins (plasma viscosity), the physico-chemical properties of the red blood cell plasma membrane (deformability) and cellular hemoglobin concentration (cytosolic viscosity), the flow velocity (aggregation), and temperature (for review see El-Sayed et al., 2005). A high blood viscosity causes a high resistance to flow, increases the power output of the heart at a given cardiac output, and might impair local blood flow.

Because of the axial migration of blood cells when blood is moved with a high velocity it has been argued that plasma viscosity is the major determinant of whole blood viscosity (Rand et al., 1970). It is determined by the concentration of plasma proteins. The effect of altered hematocrit on blood viscosity is less clear. *In vitro*, a linear relation between blood viscosity and hematocrit values between 20% and 60% has been reported, when the shear stress is low (Chien et al., 1966), which is likely due to aggregation of red blood cells (Chien et al., 1967). Aggregation varies inversely with the flow velocity (Loewe and Barbenel, 1988) and is favored by fibrogen and immune-globulins binding to the red blood cells, whereas a role for albumin is less clear (Reinhart and Nagy, 1995). The high deformability of the red blood cells facilitates blood flow even at high hematocrit, particularly in the microcirculation. In fact, improved deformability contributes to the decrease in viscosity at high shear rates (El-Sayed et al., 2005). In contrast, increased osmolality decreases deformability by an increase of internal viscosity and altered surface-to-volume ratio although effects are small in the physiological range of changes in red blood cell hemoglobin concentration (Mohandas et al., 1980). Deformability of red blood cells has a temperature optimum in the physiological range and decreases significantly at temperatures below 35°C and above 45°C (Hanss and Koutsouris, 1984), which seems to be mainly determined by the lipid composition of the plasma membrane (Heath et al., 1982), whereas variations of the intracellular hemoglobin concentration within the physiological range do not affect deformability (Mohandas and Chasis, 1993).

Exercise and training affect all of the above mentioned determinants of whole blood viscosity. There is a well-documented increase in whole blood viscosity during exercise which reverses rapidly (for review see El-Sayed et al., 2005). It is mostly due to hemo-concentration and dehydration (Platt et al., 1981; Galea and Davidson, 1985; Vandewalle et al., 1988; Geor et al., 1994; Yalcin et al., 2000). Results on exercise induced changes in the deformability of red blood cells are divergent and indicate decreased (most reports; e.g., Platt et al., 1981; Geor et al., 1994; van der Brug et al., 1995; Bouix et al., 1998; Smith et al., 2013), unchanged (Neuhaus et al., 1992), and increased deformability (Gurcan et al., 1998) (for review see El-Sayed et al., 2005). Exercise in hypoxia aggravated the adverse effect on deformability, which was associated with decreased actin and spectrin content and down-regulation of other proteins, and enhanced the response of red blood cells to oxidative stress (Mao et al., 2011). However, the exercise-induced decrease in deformability appears to be independent of oxidants produced by shear stress, because it was not prevented by strong antioxidant prophylaxis (Kayatekin et al., 2005). Studies may be hampered by the fact that PV and osmolality changes may revert rapidly depending on exercise duration and intensity indicating the proper choice of time-points for sampling when blood is collected after rather than during the exercise. Ernst and colleagues nicely report during and after exercise kinetics of blood viscosity and show that deformability of erythrocytes is increased during and normalized within a few hours after exercise (Ernst et al., 1991). The increase in blood lactate during exercise seems not to affect deformability (Simmonds et al., 2013), neither does lactate affect aggregation (Connes et al., 2007). However, there are indications that high lactate impairs deformability in untrained but improves in trained individuals (Connes et al., 2004). Training might decrease blood viscosity by improving the deformability of red blood cells. The membrane fluidity of red blood cells was increased in sprinters and long distance runners (Kamada et al., 1993), which is consistent with the finding that the exercise-induced decreased in deformability was found to be attenuated by training (Ernst, 1987; Yalcin et al., 2000). This might be explained by the higher deformability of newly formed red blood cells (Mairbäurl et al., 1983; Linderkamp et al., 1993). Erythropoietin, which was found slightly elevated (see above) seems to be favorable (Pichon et al., 2013; Zhao et al., 2013), probably by decreasing the mean red blood cell age and young red blood cells having an improved membrane flexibility (Mohandas and Chasis, 1993). In contrast, insulin-like growth factors and growth hormone seem to increase viscosity (Monnier et al., 2000; Connes et al., 2007). In summary, most studies show improved rheological properties of blood in trained individuals (see meta-analysis by Romain et al., 2011).

Together these results indicate that the increase in whole blood viscosity during exercise is caused by the combined effects of increased plasma viscosity and decreased deformability of the red blood cells, and potentially impairs microcirculation and thus O_2_ delivery to working muscle. Moderation of this effect might be brought about by NO released from endothelium and red blood cells with increased shear stress, because nitrosylation of cytoskeletal proteins in the red blood cell membrane seems to improve deformability (Grau et al., 2013). In contrast, training seems to increase deformability and to decrease whole blood viscosity in support of tissue oxygenation.

## Red blood cell mediated vasodilation

Precise control of regional blood flow is required to match substrate demand and removal of metabolites, which is of particular importance when the metabolic activity is high such as in exercising skeletal muscle. Nitric oxide (NO) is an important signaling molecule that causes local vasodilation. It is typically formed in vascular endothelial cells upon a variety of stimuli, the most important during exercise likely being shear stress (Pohl et al., 1986; Shen et al., 1995). Hemoglobin has been shown to tightly bind NO to form nitrosylhemoglobin (Hb-cys-NO; SNO-Hb) in an O_2_ saturation dependent manner with higher affinity for deoxyhemoglobin, a reaction that also causes formation of Met-Hb (Gow and Stamler, 1998; Grubina et al., 2007). Binding has been interpreted as a sink for NO produced by endothelium to prevent exaggerated and wide-spread vasodilation. However, it has also been hypothesized that Hb not only binds but also releases and/or produces NO from SNO-Hb to cause local vasodilation (Robinson and Lancaster, 2005).

It has been shown experimentally that NO released from red blood cells causes vasodilation when the shear stress is increased and when the tissue is made hypoxic (Ulker et al., 2013). Red blood cells produce bioactive NO equivalents in an O_2_ saturation, pH, and redox-state dependent manner, which appears to be an allosteric, autocatalytic reaction with characteristics of a nitrite reductase reaction (for review see Gladwin and Kim-Shapiro, 2008). When nitrite is added to fully deoxygenated Hb, NO is released and Met-Hb is formed (Gladwin and Kim-Shapiro, 2008). Bioactivity is indicated by the notion that upon nitrate infusion, NO binding to hemoglobin and vasodilation are tightly coupled and are favored by hypoxia (Crawford et al., 2006). Kleinbongard et al. (2006) presented immune-histochemical and functional evidence of the presence of an endothelial NO-synthase type of enzyme in human and mouse red blood cells indicating the potential to produce NO from L-arginine. It is unclear, however, whether this reaction is active in controlling microcirculation in working skeletal muscle (which generates a low oxygen environment because of its requirement for oxygen).

ATP in plasma is another stimulus for endothelial NO production (Sprague et al., 1996). ATP is released from many cells where it modifies a variety of functions (Praetorius and Leipziger, 2009). Local vasodilation has been shown to depended on the presence of red blood cells (Dietrich et al., 2000). Thus, it has been hypothesized that red blood cells release ATP and cause an NO-dependent increase in blood flow (Gonzalez-Alonso et al., 2002). ATP release is not only an *in vitro* phenomenon but has also been demonstrated vivo, where elevated ATP has been found in the venous effluent from exercising forearm muscle (Forrester, 1972; Ellsworth et al., 1995). This effect was even enhanced when exercise was performed in hypoxia (Gonzalez-Alonso et al., 2002).

The major stimulus for ATP release from red blood cells seems to be mechanical deformation (Sprague et al., 1996; Ellsworth et al., 2009), where ATP release seems to depend on the shear rate (Mairbäurl et al., 2013). Also *in vitro* hypoxia stimulates the release of ATP from red blood cells (Bergfeld and Forrester, 1992). Futhermore, hypoxia greatly enhances ATP release induced by shear stress indicating that effects are additive (Mairbäurl et al., 2013). Other stimulators of ATP release from red blood cells are beta adrenergic stimulators and prostacyclin (Olearczyk et al., 2001), and an increase in temperature (Kalsi and Gonzalez-Alonso, 2012). The exact release mechanism is unclear. An involvement of CFTR has been discussed (Sprague et al., 1998) but it is unclear whether CFTR is actually present in human red blood cells. A variety of other mechanisms for ATP release have been described (for review see Praetorius and Leipziger, 2009), some of which seem to involve pannexin1- (Qiu and Dahl, 2009; Qiu et al., 2011). Intravascular hemolysis seems not to contribute significantly to ATP release from erythrocytes exposed to shear-stress (Mairbäurl et al., 2013).

Taken together these results indicate that red blood cells support local vasodilation in tissues with a high O_2_ demand by directly mediating NO release and enzymatic production and by release of ATP, which causes NO release from endothelial cells by mechanisms, which are greatly enhanced in exercise when shear stress is increased by increased blood flow, O_2_ is low due to increased consumption, and the increase in temperature.

## Conclusion

**Figure 3:**
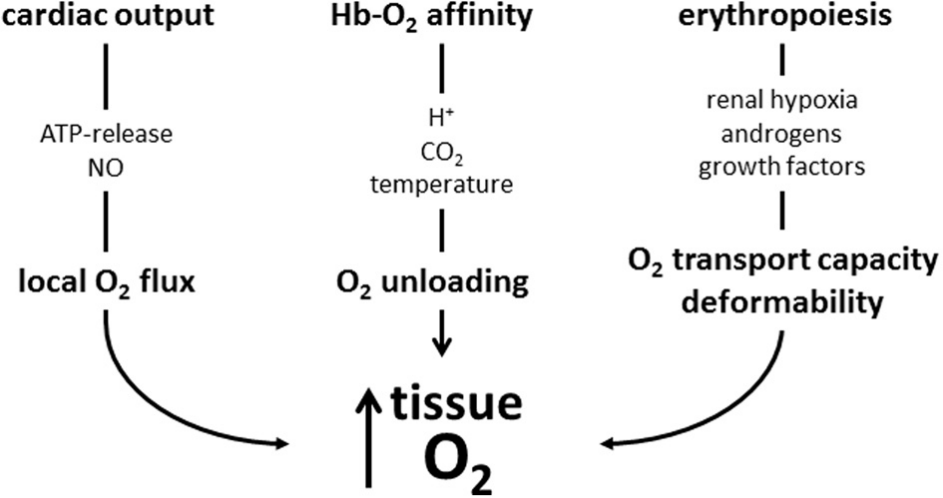
**Schematic presentation of mechanisms increasing muscle oxygen supply acutely during exercise and by training discussed in this review**. During exercise local blood flow is increased by mediators causing local vasodilation, which is supported by red blood cell-mediated NO production. Acidosis, CO_2_ and hyperthermia decrease Hb-O_2_-affinity and enhance O_2_ release from its bond to hemoglobin. These improvements may in part be blunted by increased blood viscosity (not shown in scheme). Training stimulates erythropoiesis to increase the O_2_-transport capacity. The newly formed cells also have an improved deformability which facilitates muscle blood flow. Training also increases red blood cell 2,3-DPG (not shown), which further enhances O_2_ release from Hb.

There are many mechanisms that contribute to an increased tissue oxygen supply during exercise. Figure 3 summarizes those, where red blood cell are involved. They involve adjustments during exercise and to training. During exercise the increased O_2_ demand of skeletal muscle is mainly matched by increasing muscle blood flow by increasing cardiac output, by modulating blood flow distribution among active and inactive organs, and by optimizing microcirculation (Laughlin et al., 2012). Red blood cells support local blood flow by providing the vasodilator NO by direct conversion from nitrate and by release of ATP causing endothelial NO release. At any given capillary blood flow the amount of O_2_ unloaded from Hb to the cells of working muscle can be increased greatly by decreasing Hb-O_2_ affinity. This happens as the cells enter the capillaries supplying the muscle cells, where they are exposed to increased temperature, H^+^ and CO_2_. Training further enhances O_2_ flux to the working muscle at all levels of regulation: It increases maximal cardiac output, improves blood flow to the muscles by stimulating vascularization, and improves the rheological properties of red blood cells. Training increases total hemoglobin mass by stimulating erythropoiesis, which increases the amount of O_2_ that can be carried by blood. It also increases red blood cell 2,3-DPG, which increases the sensitivity of Hb-O_2_ affinity to acidification dependent O_2_-release. The system appears to be optimized for exercise at low altitude, because in an hypoxic environment the decreased arterial PO_2_, which is the major determinant for O_2_ diffusion, cannot be compensated adequately by the above mentioned O_2_ transport mechanisms resulting in a decrease in performance with increasing degree of hypoxia (Cerretelli and DiPrampero, 1985).

### Conflict of interest statement

The author declares that the research was conducted in the absence of any commercial or financial relationships that could be construed as a potential conflict of interest.
